# IL-17A, IL-17E and IL-17F as Potential Biomarkers for the Intensity of Low-Grade Inflammation and the Risk of Cardiovascular Diseases in Obese People

**DOI:** 10.3390/nu14030643

**Published:** 2022-02-02

**Authors:** Ewelina Polak-Szczybyło, Jacek Tabarkiewicz

**Affiliations:** 1 Department of Dietetics, Institute of Health Sciences, Medical College of Rzeszow University, University of Rzeszow, 35-959 Rzeszow, Poland; epolak@ur.edu.pl; 2 Department of Human Immunology, Institute of Medical Sciences, Medical College of Rzeszow University, University of Rzeszow, 35-959 Rzeszow, Poland; 3 Laboratory for Translational Research in Medicine, Centre for Innovative Research in Medical and Natural Sciences, Medical College of Rzeszow University, University of Rzeszow, 35-959 Rzeszow, Poland

**Keywords:** obesity, low-grade inflammation, IL-17A, IL-17E, IL-17F, metabolic syndrome, cardiovascular disease

## Abstract

Low-grade inflammation is a factor that predisposes to many obesity-related comorbidities. The immune mechanisms controlling the inflammatory response related to the secretory activity of adipocytes and its consequences for the organism are still under investigation. Methods: 84 obese adult volunteers (BMI ≥ 30 kg/m^2^) were tested by BIA. Serum samples were collected to analyze the concentrations of interleukins IL-17A, IL-17E and IL-17F. The subjects completed the original questionnaire, the FFQ-6 food consumption frequency questionnaire and the food diary. Results: The level of IL-17E and IL-17F was positively correlated with the BMI value and the level of IL-17E increased with the content of subcutaneous fat. Its increased blood concentration was also observed in individuals who declared that they were diagnosed with atherosclerosis and/or were taking beta-blockers. Products that were related with a low level of the above-mentioned interleukins were vegetables, groats, eggs, red meat, fast-food and alcohol. The level of these interleukins was positively correlated with the frequent consumption of confectionery and breakfast cereals. Nutrients that decreased the concentrations of IL-17 isoforms were potassium, iron, vitamins B6 and C, and folic acid. Conclusions: Both IL-17E and IL-17F may be closely related to the intensity of low-grade inflammation and be biomarkers of cardiovascular disease risk. Food products or the nutrients they contain may affect the levels of the above-mentioned interleukins as well as IL-17A.

## 1. Introduction

Overweight and obesity are the result of the excessive accumulation of adipose tissue as a consequence of increased energy consumption as food in relation to the body’s energy needs [1]. According to the World Health Organization, in 2016, there were 39.1% over-weight people (body mass index (BMI) ≥ 25 kg/m^2^) and 13.1% obese (BMI ≥ 30 kg/m^2^) worldwide in 2016. More accurate data depends largely on the region, e.g., in Europe in 2016 23.3% of people had obesity, and in the Americas, 28.6% [2]. It is estimated that in 2030, over 51% of the world’s population will be obese [3]. Excessive adipose tissue is not only an aesthetic issue, but mainly a health problem contributing to the occurrence of metabolic syndrome and many diseases such as: type 2 diabetes, cardiovascular diseases, cancers and autoimmune diseases [4]. Both animal and human studies have confirmed that these diseases and obesity are linked to low-grade inflammation caused by the infiltration of adipose tissue by immune cells. This is evidenced by the increased level of acute phase proteins and pro-inflammatory mediators in the blood serum of obese individuals [5]. Inflammatory markers such as CRP, IL-6 or TNF-α are the best studied. However, there is little information considering the IL-17 family interleukins in correlation with obesity. These interleukins are produced mainly by Th17 lymphocytes, which are physiologically involved in the neutralization of fungal and bacterial pathogens by activating neutrophils. It turns out, however, that they can also play a key role in the emergence and progression of cancer and chronic inflammatory diseases in which obesity is considered a risk factor [1,6]. The IL-17 family in humans comprises IL-17 6 isoforms from A to F. It has been recently suggested in studies that IL-17A is involved in the induction of low-grade inflammation and adipogenesis, and also affects blood glucose levels [7]. The cytokines of the IL-17 family are released not only by Th-17 lymphocytes, but also by other immune cells, e.g., neutrophils, mast cells and ILCs, which are residing in many tissue. The pleiotropic effect of IL-17 influences neutrophils and macrophages as well as many cells outside the immune system: endothelial cells, fibroblasts, osteoclasts, chondrocytes, osteoblasts and keratinocytes [8]. The other member of this family, IL-17C, is mainly produced by epithelial cells and was recently found to be important in promoting cytokines and anti-microbial peptides production in the gastrointestinal tract [9]. L-17E mediates a positive feedback loop between epithelial and immune cells in the gut mucosa, leading to the augmentation of Th_2_ response to external cues (commensal flora, infections, etc.) [10].

Obesity is known to predispose to autoimmune and inflammatory disorders, and studies most often describe the Th-17 cells, IL-17A and IL-17E in the course of diseases such as bronchial asthma, obstructive pulmonary disease, multiple sclerosis, systemic lupus erythematosus, inflammatory bowel disease and psoriasis [11,12,13,14,15,16]. Therefore, it is important to know and understand the relationship between obesity and members of the IL-17 family. There is still little research into this topic. It is presumed that the secretion of IL-17 depends largely on the level of IL-6 being present in higher concentrations in the serum of obese subjects. IL-6 is necessary for the differentiation of naive CD4^+^ T cells into the Th17 lineage [17]. Additionally, in a mouse model study, the low level of leptin was associated with excessive food consumption followed by exacerbated inflammation stimulated by the secretion of IL-6, and at the same time an increased level of IL-17A [18]. Although Th17 cells are believed to be responsible for the production of the IL-17 family’s interleukins, there are speculations that they may also be secreted by other cells. In studies, the neutralization of IL-17A in obese mice suppressed neutrophil recruitment, correlating with decreased levels of neutrophil reconstructing chemokines such as CXCL_1_ and CXCL_2_ [18,19].

Taking into account the negligible amount of research focused on the level of IL-17 family interleukins in the relation to low-grade inflammation, the aim of this study was to determine whether these interleukins can be a predictor of cardiovascular disease and to identify dietary factors influencing their level.

## 2. Materials and Methods

### 2.1. Ethics

The study was conducted at the Center for Innovative Research in Medical and Natural Sciences, College for Medical Sciences of the University of Rzeszow (Poland). The research project was approved by the institutional Bioethics Committee at the University of Rzeszow (Resolution No. 10/04/2017) and was carried out in accordance with the Helsinki Declaration. Each person participating in it signed voluntarily and consciously consent to participate in the study.

### 2.2. Subjects

**Figure 1:**
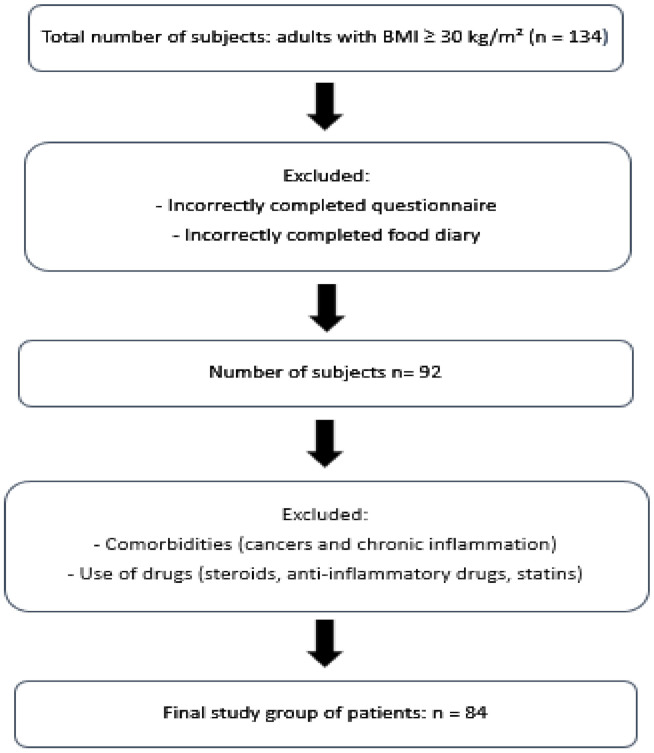
Flow chart demonstrating study’s participants’ selection.

Due to the BIA examination procedure, the project did not involve pregnant women, people with epilepsy, cardiac pacemakers, implants and prostheses. One hundred and thirty-four volunteers with BMI equal to or greater than 30 kg/m^2^ were examined. The study excluded individuals whose questionnaires and food diaries were incomplete, and those whose disease entities (cancer, autoimmune diseases and chronic inflammation) as well as medications (steroids, anti-inflammatory drugs, statins) could change the level of interleukins. The Figure 1 shows the process of inclusion or exclusion into the study group.

The 84 volunteers were included to the study group, including 62 women (73.8%) and 22 men (26.2%). The age ranged from 18 to 73 and the mean age was 41.38 ± 12.54 years. The average body weight of the examined persons was 103.01 ± 18.23 kg and ranged from 70.6 kg to 156.4 kg. The height of the participants ranged from 149 cm to 192 cm, and their average height was estimated at 167.44 ± 9.01 cm. The BMI of the examined individuals ranged from 30.0 to 58.1 kg/m^2^, averaging 36.65 ± 5.27 kg/m^2^. Obesity of the 1st degree was diagnosed in 40.5% of cases, and 2nd and 3rd degree obesity in 40.5% and 19%, respectively. The Table 1 presents diseases declared by participants and medications taken by them.

### 2.3. Blood Collection and Analysis of Interleukins and Biochemical Parameters

Blood sampling was performed in the morning (7: 00 AM–9: 00 AM) by a qualified person. The subjects were fasted, with a 24-h interval from the consumption of alcohol, coffee, diuretics and from the last physical activity. Blood samples were drawn to MLVacuCol^®^ (MEDLAB-PRODUCTS Sp. z o.o., Raszyn, Poland) from antecubital vein and were centrifuged at 700× *g* and room temperature. The serum was cryopreserved in −86 °C until analysis with commercially available kit Milliplex MAP Human Th17 Magnetic Bead Panel Kit magnetic bead multiplex assay method (Merck Millipore, Burlington, MA, USA) and FLEXMAP 3D^®^ System (Merck Millipore, Burlington, MA, USA).

### 2.4. Anthropometric Measurements and Body Composition Analysis Using the BIA Method

Anthropometric measurements and BIA examinations were performed in the morning on an empty stomach. Body height was measured twice with a Tanita HR-001 height measuring device (TANITA, Tokyo, Japan) and the mean was calculated. The body composition analysis was performed using the electric bioimpedance method, using the MC 780 MA analyzer (TANITA, Tokyo, Japan) with variable frequencies: 5 kHz, 50 kHz and 250 kHz. The acquired parameters were fat mass (FM), fat free mass (FFM), muscle mass (MM), total body water (TBW), intra- and extra-cellular water (ICW and ECW) (kg and %) and visceral adipose tissue (VF) (level). The above parameters were also obtained in a segmental approach (right leg, left leg, torso, right hand, left hand). The results of the analysis were collected using the GMON PRO software (Medizin & Service, Chemnitz, Germany).

### 2.5. Lifestyle and Nutrition Assessment

The respondents completed the original questionnaire (File S1) enriched with a standardized FFQ-6 food consumption frequency questionnaire. Additionally, intake of anti-inflammatory products was analyzed. In addition, before the study, each participant completed a nutritional diary containing 3 days (2 working days, 1 weekend) of normal nutrition according to detailed instructions. Nutritional values were calculated using the Aliant diet program (Cambridge Nutritional Sciences, Alva, UK). The program used Polish, American, Swiss, Norwegian, French and German databases of the nutritional value of products. The information obtained was related to the amount of: kilocalories, proteins, amino acids (endogenous and exogenous), fats (fatty acids: saturated, unsaturated: polyunsaturated, monounsaturated, trans), digestible carbohydrates (starch, maltose, lactose, glucose, sucrose, fructose), non-digestible carbohydrates (fiber), vitamins, minerals, beta carotene, alcohol, average glycemic index (GI), average glycemic load (GL), and PRAL (Potential Renal Acid Load).

### 2.6. Statistical Analysis

The statistical analysis of the data was performed using the Statistica 13.1 software (StatSoft, Krakow, Poland). Non-parametric tests were performed due to the failure to meet the basic assumptions of the parametric tests (compliance of the distributions of the examined variables with the normal distribution). The verification was performed using the Shapiro–Wilk W test. Mann–Whitney’s U test was used to assess the differences in the average level of a numerical feature in two populations, and the Spearman’s rank correlation coefficient was used to determine the correlation of two variables that did not meet the criterion of normal distribution. The relationships of qualitative variables were assessed using the Pearson chi-square test. The level of statistical significance was set to *p* < 0.05.

## 3. Results

**Figure 2:**
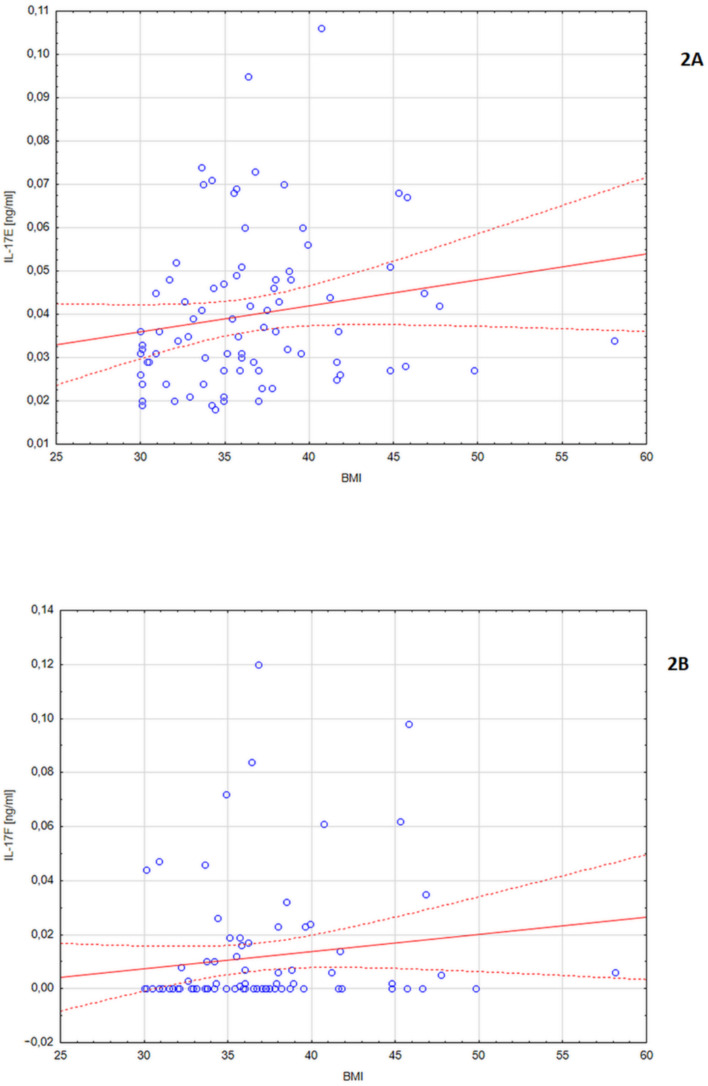
(**A**) Distribution of IL-17E values depending on the BMI level (r = 0.24, *p* = 0.026); (**B**) distribution of IL-17F values depending on the BMI level (r = 0.25, *p* = 0.025).

Subjects with a higher BMI had a higher level of IL-17F (r = 0.25, *p* = 0.025) and IL-17E (r = 0.24, *p* = 0.026) (Figure 2). A positive correlation was found between the level of IL-17F and the content of adipose tissue in the right leg, expressed in % (r = 0.27, *p* = 0.015) and in kg (r = 0.25, *p* = 0.025), in the left leg, expressed in % (r = 0.24, *p* = 0.028) and in kg (r = 0.24, *p* = 0.031), in the right hand, expressed in % (r = 0.30, *p* = 0.006) and in kg (r = 0.22, *p* = 0.049), and in the left hand, expressed in % (r = 0.27, *p* = 0.013).

The level of IL-17E was also higher in the case of a high content of adipose tissue in the right leg, expressed in % (r = 0.25, *p* = 0.023) and in kg (r = 0.22, *p* = 0.043) (Supplementary Table S1).

**Figure 3:**
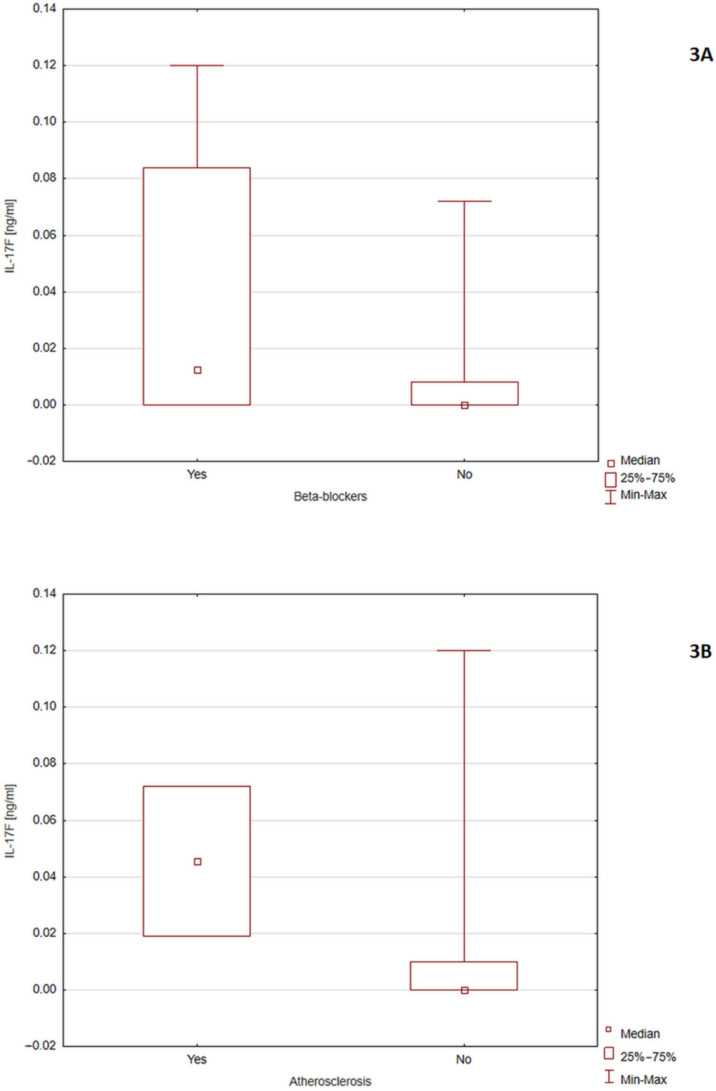
(**A**) comparison of IL-17F levels in people with and without atherosclerosis (*p* = 0.04) and (**B**) people using and not taking beta-blockers (*p* = 0.024).

Subjects diagnosed with atherosclerosis (*p* = 0.04) and taking beta-blockers (*p* = 0.024) had higher levels of IL-17F (Figure 3).

Individuals who often consumed confectionery bread showed a higher level of IL-17A (r = 0.22, *p* = 0.046), while the respondents who often consumed confectionery bread (r = 0.25, *p* = 0.020) and breakfast cereals (r = 0.28, *p* = 0.011) had a higher level of IL -17F. The frequent consumption of groats (buckwheat, barley, millet) (r = −0.25, *p* = 0.023), eggs (r = −0.22, *p* = 0.043) and vegetables (r = −0.25, *p* = 0.026) suppressed the level of IL-17A. Frequent consumption of fast-food dishes lowered the level of IL-17E (r = −0.22, *p* = 0.046), and the level of IL-17F was negatively correlated with the frequent consumption of red meat (r = −0.27, *p* = 0.014), and alcohol (r = −0.22, *p* = 0.045) (Supplementary Table S2). After summarizing the nutrients contained in the nutritional diaries and subjecting the data to statistical analysis, the food components influencing the level of IL-17E and IL-17F were distinguished. The higher the PRAL ratio of the diet (r = 0.25, *p* = 0.023), the higher the level of IL-17E was in the blood of the subjects. The high PRAL ratio reflects the high content of acid-forming substances in the diet. On the other hand, the level of IL-17F was lower in people whose diets contained higher amounts of potassium (r = −0.30, *p* = 0.005), iron (r = −0.22, *p* = 0.042), vitamin B6 (r = −0.23, *p* = 0.036)), vitamin C (r = −0.28, *p* = 0.009) and folic acid (r = −0.24, *p* = 0.030) (Supplementary Table S3).

## 4. Discussion

Obesity increases the likelihood of developing diseases such as rheumatoid arthritis, multiple sclerosis, psoriasis and cancers. The cytokines of the IL-17 family play an important role in the pathogenesis of all these diseases [1,20,21,22,23]. Increased concentrations of IL-17F have been reported in skin sections of psoriasis patients, and obesity is known to be a risk factor for the development of this disease [24]. We found a positive correlation in the subjects between BMI and the level of IL-17E and IL-17F cytokines. We have also shown that the level of IL-17F increased with the amount of subcutaneous fat in the limbs. Considering these relationships, it is worth mentioning that so far there is a small number of studies focused on the level of IL-17E and IL-17F in the obese individuals. The study of Glatt S. et al. supports hypothesis that IL-17F plays a crucial role in chronic tissue inflammation and authors confirmed this in a preclinical model as well as in a placebo-controlled proof-of-concept (PoC) clinical trial randomized patients with psoriatic arthritis (PsA) to bimekizumab [25]. IL-17A is also referred to as IL-17 and has so far been the most studied interleukin in this group in correlation with obesity and the metabolic syndrome. Obesity promotes the expansion of Th17 cells and subsequent IL-17 production exacerbating disease in murine models of autoimmunity, such as EAE and colitis [17]. In diet-induced obese (DIO) mice, Th17 cell pools are expanded and they produce progressively more IL-17 than lean littermates, in what has been shown to be an IL-6-dependent process [26]. The leptin deficiency could enhance the IL-17 dependent inflammatory process in the mouse model [27]. On the other hand, hyperleptinemia is associated with the progression of atherosclerosis through the secretion of pro-inflammatory cytokines, including IL-17 and other cytokines, to promote chronic inflammation and obesity-associated metabolic syndrome [28]. The other detrimental effect of the release of IL-17 in adipose tissue is long-term reduced insulin sensitivity, which could be responsible for the development of diabetes mellitus type II as a part of metabolic syndrome [29,30].

Similarities exist between the immunopathologies of psoriasis, atherosclerosis and metabolic syndrome. This includes promoting the differentiation of Th1 and Th17 cells and the secretion of pro-inflammatory cytokines such as TNF-α, IFN-γ, IL-17A and IL-22 [31]. In atherosclerosis, IL-17 and TNF-α synergistically activate NF-κB signaling and mitogen-activated protein kinases to induce neutrophil-attracting chemokines and other inflammation modulators, and increase aortic inflammation and thrombosis, eventually exacerbating cardiovascular diseases and increasing the morbidity associated with them [31]. The neutralization of IL-17 lead to a significant improvement in patients with psoriasis or ankylosing spondylitis including lasso cardiovascular comorbidities [32,33]

Bertol et al. found that the expression of IL-17A in the vascular fraction of the adipose tissue stroma was higher in overweight and obese patients compared to the control group with a normal body weight, contrary to the earlier opinion that IL-17A counteracts adipogenesis [34]. A study in a mouse model by Zuñiga et al. showed that the expression of IL-17A by γδ T lymphocytes is higher in subcutaneous adipose tissue, just as IL-17F was present in higher concentrations in individuals with a high content of subcutaneous tissue [7]. The excess of adipose tissue is a factor predisposing to metabolic syndrome, which could result in cardiovascular diseases, and the level of IL-17F is associated with atherosclerotic processes [35]. This finding is consistent with the positive correlation in our own studies between high IL-17F levels and the occurrence of atherosclerosis or treatment with beta-blockers used in these patients. Dalmas et al. showed that in obese patients, especially those with type 2 diabetes, there was an increased level of IL-17A in the adipose tissue compared to people with a normal body weight [24]. In addition, blocking the action of IL-17A resulted in a reduction in the intensity of inflammation in obese patients with a non-alcoholic fatty liver (NASH). Hence, it can be concluded that the concentration of IL-17A in the blood may be a predictor of cardiovascular disease or metabolic syndrome [24].

Diet is an undeniably important factor in the prevention of obesity and civilization diseases. It may be related to the influence of particular products or nutrients on the intensity of low-grade inflammation. Our analysis showed a positive correlation between the level of IL-17A and the frequency of consumption of pro-inflammatory food products, such as confectionery, and a negative correlation with products with anti-inflammatory effects, such as vegetables, cereals and eggs. Sweet confectionery bread contains both sugar (sucrose) and palm oil (rich in palmitic acid). These components have been shown to generate inflammation through IL-17A [36,37]. The polyphenols in vegetables have the opposite effect, reducing IL-17A [38]. Polyphenols include phenolic acid, flavonoids and anthocyanins in vegetables, and their anti-inflammatory properties have been thoroughly studied [39,40]. Frequent eggs consumption correlated with lower IL-17A levels. Ingredients that may exhibit anti-inflammatory properties of eggs are ovotransferrin, lysozyme, some proteins, phospholipids, lutein and zeaxanthin [41,42,43]. The frequent consumption of products such as vegetables, fruit, eggs, groats (e.g., buckwheat, barley, millet) lowered the concentration of IL-17A in a study on about 700 children with asthma [44]. There are many studies related to the influence of diet on the concentration of IL-17A, and few related to the level of IL-17E or IL-17F in correlation with eating habits [45]. Our own research has shown a lower level of IL-17F in people who drink alcohol frequently. There are no studies that can be addressed regarding interleukins 17E and 17F, but there are studies supporting the suppressive effect of alcohol on IL-17A secretion [46,47,48]. IL-17E negatively correlated with the consumption of fast-food dishes. This may be due to the high content of fats in these foods, in particular saturated fatty acids and trans isomers, which, by increasing inflammation, contribute to the development of metabolic diseases [49]. IL-17E levels were decreased in a fatty liver study in mice as a result of a high-fat diet. It was assumed that this is due to the property of IL-17E, which prevents the accumulation of fat in the liver [50]. In the authors’ own research, the level of IL-17E was lowered by the frequent consumption of red meat, which has not been researched yet. However, an iron deficiency in the diet may be associated with inflammation and, at the same time, with an increase in the level of IL-6, which may affect the level of interleukins from the IL-17 family [51]. Research has shown that food ingredients such as potassium, vitamin B6, vitamin C, and folic acid reduce IL-17F levels. These ingredients are largely found in foods considered healthy with anti-inflammatory properties, such as the ones mentioned earlier [44]. It is worth mentioning the PRAL coefficient, the high value of which was positively correlated with the level of IL-17E. Food PRAL is a factor that determines the acid- or alkaline-forming properties of food in the human body, which largely reflects the content of sulfur amino acids, phosphorus, magnesium, potassium and calcium in foods [52]. The correlation of the ratio and level of interleukins from the 17 family has not been studied so far, but the literature shows that products with a high PRAL have a pro-inflammatory effect and their consumption is associated with obesity and / or type 2 diabetes [53,54,55].

Despite the innovative nature of this study, it has limitations such as the small number of respondents and the lack of a control group. It should be additionally noted that a limitation may also be the careless completion of the nutritional diary by the respondents, or the subjective assessment of the frequency of consumption of certain products in the FFQ-6 sheet. Nevertheless, these studies have shown dependencies that should be explored in future experiments, as they reveal the mechanisms linking low-grade inflammation with heart disease and diet, which is important for both prevention and pharmacotherapy of these diseases.

## 5. Conclusions

The serum concentrations of IL-17E and IL-17F increases with the value of the BMI. In addition, the concentration of IL-17F is higher in people with a higher body weight and higher content of adipose tissue. Based on our own results and analysis of available data, we suggest that both of them may be predictors of the severity of low-grade inflammation and the risk of heart disease. It is important to be able to create an appropriate dietary intervention based on this research. The concentration of interleukins from the IL-17 family is increased by acid-forming products (high PRAL ratio), confectionery and breakfast cereals. The concentration of these interleukins is suppressed by high consumption of vegetables, groats (buckwheat, millet, barley), eggs and red meat. This may confirm the thesis that the nutrients contained in them exhibit anti-inflammatory properties. The exception is fast-food, where elevated IL-17E may prevent the accumulation of fat in the liver. The level of IL-17F is suppressed by the high content of potassium, iron, vitamin B6, folic acid and vitamin C in the diet, which makes it possible to carry out appropriate supplementation or diet therapy in people at risk of metabolic syndrome. All of the above-mentioned conclusions require further research.

## Figures and Tables

**Table 1:** Diseases and medications declared by participants.

Diagnosed Chronic Diseases *	**n**	**%**
Hypertension	21	25.0%
Fatty liver	3	3.6%
Treated hypothyroidism	6	7.1%
Atherosclerosis	2	2.4%
Drugs Taken *	**n**	**%**
Antihypertensive drugs (excluding beta-blockers)	21	25.0%
Anticoagulants	4	4.8%
Levothyroxine	4	4.8%
Beta blockers	10	11.9%
NSAIDs	3	3.6%

n—number of observations; %—percentage of the studied group; * possibility to indicate multiple answers.

## Data Availability

The data presented in this study are available upon request from the corresponding author. The data are not publicly available, as they include sensitive clinical data.

## Supplementary Materials

The following supporting information can be downloaded at: https://www.mdpi.com/article/10.3390/nu14030643/s1. Table S1: Correlation between parameters obtained from body composition analysis and cytokine levels, Table S2: Correlation of the relationship between the frequency of consumption of selected products and the level of cytokines, Table S3: Results of the statistical analysis between the frequency of nutrient consumption and the level of cytokines, File S1: Original food consumption frequency questionnaire.
