# Supplementary material for: IL-17A, IL-17E and IL-17F as Potential Biomarkers for the Intensity of Low-Grade Inflammation and the Risk of Cardiovascular Diseases in Obese People
Source: Nutrients. 2022 Feb 2;14(3):643. doi: 10.3390/nu14030643 (PMC8839991; doi:10.3390/nu14030643)
Supplement: Supplementary file 1 [file nutrients-14-00643-s001.zip › nutrients-1567403-supplementary.pdf]

Table S1. Correlation between parameters obtained from body composition analysis and cytokine levels. Spearman's Rank correlation coefficient is listed in the columns and for statistically significant correlations it is followed by p value.

Parameters	IL-17A [pg/ml]	IL-17E [ng/ml]	IL-17F [ng/ml]
Height [cm]	0.11	-0.12	-0.25* (p=0,027)
Body weight [kg]	0.18	0.08	0.01
BMI [kg/m²]	0.14	0.24*(p=0,026)	0.25* (p=0,025)
Fat tissue [%]	0.09	0.11	0.15
Fat tissue [kg]	0.11	0.10	0.08
Visceral fat level	-0.01	0.16	0.04
Lean body mass [kg]	0.19	0.03	-0.08
Total water content [kg]	0.19	0.03	-0.08
Muscle mass [kg]	0.19	0.03	-0.08
Bone weight [kg]	0.19	0.03	-0.08
Right leg adipose tissue [%]	0.05	0.25* (p=0,023)	0.27* (p=0,015)
Right leg adipose tissue [kg]	0.18	0.22* (p=0,043)	0.25* (p=0,025)
Right leg lean body mass [kg]	0.15	0.00	-0.07
Right leg muscle mass [kg]	0.15	0.00	-0.07
Left leg adipose tissue [%]	0.06	0.21	0.24* (p=0,028)
Left leg adipose tissue [kg]	0.17	0.21	0.24* (p=0,031)
Left leg lean body mass [kg]	0.13	0.02	-0.06
Left leg muscle mass [kg]	0.13	0.02	-0.06
Right hand adipose tissue [%]	0.09	0.19	0.30* (p=0,006)
Right hand adipose tissue [kg]	0.18	0.17	0.22* (p=0,049)
Right hand lean body mass [kg]	0.16	0.03	-0.13
Right hand muscle mass [kg]	0.16	0.04	-0.13
Left hand adipose tissue [%]	0.11	0.19	0.27* (p=0,013)
Left hand adipose tissue [kg]	0.16	0.17	0.20
Left hand lean body mass [kg]	0.16	0.03	-0.10
Left hand muscle mass [kg]	0.17	0.04	-0.10
Torso adipose tissue [%]	-0.05	-0.02	-0.09
Torso adipose tissue [kg]	0.06	0.02	-0.10
Torso lean body mass [kg]	0.20	0.05	-0.07
Torso muscle mass [kg]	0.21	0.05	-0.07

* and red color indicate significant values ($p < 0.05$)

Table S2. Correlation of the relationship between the frequency of consumption of selected products and the level of cytokines. Spearman's Rank correlation coefficient is listed in the columns and for statistically significant correlations it is followed by p value.

**Products**	**IL-17A [pg/ml]**	**IL-17E [ng/ml]**	**IL-17F [ng/ml]**
White bread	0,09	-0,10	-0,05
Wholemeal bread	0,01	0,06	0,07
Confectionery bread	0,22* (p=0,046)	0,16	0,25* (p=0,020)
Oat flakes, barley, rye	-0,00	0,06	0,28* (p=0,011)
Buckwheat, barley, millet	-0,25* (p=0,023)	-0,13	0,07
White rice	0,11	-0,10	-0,12
Brown rice	-0,09	-0,01	0,19
White noodles	0,00	-0,07	0,03
Wholemeal pasta	-0,19	0,08	0,09
Potatoes	-0,17	-0,18	-0,11
Poultry	-0,04	-0,11	0,07
Red meat	-0,07	-0,18	-0,27* (p=0,014)
Fish	-0,04	0,10	-0,10
Cold cuts, frankfurters, sausages	-0,14	0,02	-0,19
Milk	-0,07	-0,04	-0,00
Natural sour milk products	-0,10	0,02	0,05
Sour fruit milk products	0,02	0,08	0,20
Cottage cheese	-0,06	0,11	0,04
Cheese	-0,00	0,14	-0,03
Eggs	-0,22* (p=0,043)	-0,01	-0,12
Vegetables	-0,25* (p=0,026)	-0,05	0,00
Fruit	-0,11	0,06	0,07
Cakes, cookies	-0,11	-0,11	-0,06
Bars, chocolates, chocolates	-0,12	-0,02	0,06
Juices, nectars, fruit drinks	0,12	0,05	0,10
Sweetened carbonated drinks	-0,02	-0,11	0,00
Alcohol	-0,01	-0,18	-0,22* (p=0,045)
Fast-food dishes	-0,04	-0,22*(p=0,046)	-0,10

* and red color indicate significant values ($p < 0.05$)

Table S3. Results of the statistical analysis between the frequency of nutrients consumption and the level of cytokines. Rank correlation coefficient is listed in the columns and for statistically significant correlations it is followed by p value.

**Nutrients**	**IL-17A [pg/ml]**	**IL-17E [ng/ml]**	**IL-17F [ng/ml]**
Energy [kcal]	0,05	0,04	-0,04
Protein [g]	-0,01	0,02	-0,18
Fat [g]	-0,01	-0,03	-0,05
Digestible carbohydrates [g]	0,07	0,03	0,01
Fiber [g]	-0,11	-0,02	-0,12
Vegetable protein [g]	0,04	-0,03	-0,09
Animal protein [g]	0,03	0,10	-0,05
Sugars [g]	0,11	0,13	0,20
Fructose [g]	-0,02	0,12	0,04
Galactose [g]	-0,01	0,00	0,14
Glucose [g]	-0,04	0,10	0,04
Lactose [g]	-0,09	-0,10	0,02
Maltose [g]	0,05	0,05	0,13
Sucrose [g]	0,13	0,09	0,21
Starch [g]	0,01	0,05	-0,01
SFA [g]	-0,11	-0,03	-0,01
MUFA [g]	0,01	0,02	-0,03
n-3 fatty acids [g]	-0,05	0,15	0,18
n-6 fatty acids [g]	0,11	-0,01	-0,11
PUFA [g]	0,09	-0,01	-0,09
Cholesterol [mg]	-0,02	0,03	-0,07
Trans-fatty acids in total [g]	0,02	-0,03	-0,05
Sodium [mg]	-0,07	0,03	-0,11
Salt [g]	-0,07	0,03	-0,10
Potassium [mg]	-0,19	-0,19	-0,30* (p=0,005)
Calcium [mg]	-0,07	-0,18	-0,14
Phosphorus [mg]	-0,01	-0,06	-0,15
Magnesium [mg]	-0,07	-0,15	-0,17
Iron [mg]	-0,06	-0,05	-0,22* (p=0,042)
Zinc [mg]	-0,01	0,04	-0,08
Copper [mg]	-0,08	-0,13	-0,18
Manganese [mg]	-0,03	0,00	-0,03
Selenium [µg]	0,12	-0,15	0,01
Iodine [µg]	-0,07	-0,09	-0,18
Vit. A [µg]	-0,14	-0,01	-0,08
Retinol [µg]	0,01	-0,00	-0,08

Beta carotene [μg]	-0,17	-0,05	-0,09
Vit. D [μg]	-0,07	0,09	-0,17
Vit. E [mg]	0,07	-0,08	-0,11
Vit. K [μg]	-0,05	-0,11	-0,10
Vit. B1 [mg]	-0,13	-0,00	-0,12
Vit. B2 [mg]	-0,03	-0,07	-0,09
Vit. B3 [mg]	-0,10	-0,02	-0,17
Vit. B6 [mg]	-0,14	-0,08	-0,23* (p=0,036)
Folates [μg]	-0,06	-0,08	-0,24* (p=0,030)
Vit. B12 [μg]	-0,09	0,03	-0,14
Vit. C [mg]	-0,13	-0,11	-0,28* (p=0,009)
Isoleucine [mg]	0,03	0,11	-0,09
Leucine [mg]	0,03	0,12	-0,09
Lysine [mg]	-0,00	0,11	-0,09
Methionine [mg]	0,04	0,13	-0,07
Cystine [mg]	0,06	0,16	-0,09
Phenylalanine [mg]	0,01	0,10	-0,09
Tyrosine [mg]	0,03	0,08	-0,10
Threonine [mg]	0,01	0,11	-0,09
Tryptophan [mg]	0,01	0,09	-0,09
Valine [mg]	0,01	0,08	-0,10
Arginine [mg]	-0,02	0,11	-0,10
Histidine [mg]	-0,01	0,13	-0,11
Alanine [mg]	0,01	0,13	-0,10
Kw. aspartic [mg]	-0,04	0,04	-0,15
Kw. glutamic [mg]	0,02	0,11	-0,09
Glycine [mg]	-0,05	0,12	-0,08
Proline [mg]	0,06	0,15	-0,04
Serine [mg]	0,03	0,10	-0,10
GI	-0,02	-0,13	-0,19
GL	0,06	0,00	0,04
PRAL	0,21	0,25* (p=0,023)	0,16

* and red color indicate significant values ($p < 0.05$)indicate significant values ($p < 0.05$).

File S1 Original food consumption frequency questionnaire.

Diet and lifestyle survey:

Given name and surname:

1. Sex
   a) female
   b) male

2. Age …… years

3. Body weight ………… kg

4. Body height ……… cm

5. Place of residence

   a) rural
   b) urban

6. Assess your financial condition:
   a) very poor
   b) poor
   c) good
   d) very good

7. Diagnosed chronic diseases?
   ………......................
   ....

8. Medication?
   ……
   …………………………………………………………………………………

9. Diet supplements?
   ……
   …………………………………………………………………………………

10. Are you drinking tea?
    a) yes
    b) no

11. If you answered yes in question 10, please describe what type of tea are you drinking and how frequent?
    ……
    …………………………………………………………………………………

12. Are you drinking coffee?
    a) yes
    b) no

13. Are you use sugar for sweetening tea or coffee?
    a) yes

b) no

14. Are you drinking water?
    a) yes
    b) no

15. How much fluid do you consume daily?
    a) <0,5 liter
    b) 0,5-1 liter
    c) 1-2 liters
    d) > 2 liters

16. Are you drinking alcohol?
    a) yes
    b) no

17. Are you salting foods and/or beverages?
    a) yes
    b) no

18. Are you using seasonings: Vegeta, Kucharek czy Maggi?
    a) yes
    b) no

19. How many meals do you eat per day?
    a) 1-2
    b) 3
    c) 4-5

20. Do you eat between meals?
    a) yes
    b) no

21. What are the meal times?
    ……
    ………………………………………………………………………………………

22. How often do you eat these products? (mark X)

Products	Every day	4-5 times per week	2-3 times per week	once per week	1-2 times per month	I am not eating ths product at all
White (e.g. wheat) bread						
Dark (e.g. rye, gra-ham) bread						
Confectioneries e.g. donuts, buns, crois-sants						
Oat, barley and rye flakes						

Buckwheat, barley, millet						
White rice						
Brown rice						
Wheat noodles						
Not wheat noodles						
Potatoes						
Poultry (chicken, turkey)						
Red meat (pork, beef, lamb)						
Fishes						
Cold cuts, sausages, etc.						
Milk						
Sour natural milk products (butter-milk, kefir, yoghurt)						
Sour fruit milk products (butter-milk, kefir, yoghurt)						
White cheese e.g. cottage cheese						
Cheese						
Eggs						
Vegetables						
Fruits						
Cakes						
Chocolate bars, chocolates, etc.						
Fruit juices, nectars, fruit drinks						
Sweetened car-bonated drinks e.g. Coke, Pepsi, Mirinda, Fanta, or-angeade						
Alcohol						
Fast-food						

In the questions below you can choose more than one answer.

23. Where do you eat your meals?

a) at home
b) at work
c) in restaurants, canteens or fast foodzie spożywa Pan/Pani posiłki?

24. Which fats do you use for frying?

a) olive oil
b) sunflower oil
c) rapeseed oil
d) lard
e) butter

25. What do you use to spread your sandwiches?

a) butter
b) margarine
c) olive oil
d) fromage

26. What do you use for salads?
a) olive oil
b) rapeseed or sunflower oil
c) mayonnaise
d) yogurt

27. What are the most common heat treatment methods in your kitchen?
a) cooking
b) choking
c) frying
d) baking

28. Do you smoke cigarettes?

a) yes
b) no

29. Do you play sports??
a) yes
b) no

30. If you answered yes in question 29, please indicate what type of sport?
……
……………………………………………………………………………………
